# Hemostatically Active Proteinase Produced by *Aspergillus ochraceus*: Key Specific Properties and Effect on Target Proteins

**DOI:** 10.3390/ijms241813870

**Published:** 2023-09-08

**Authors:** Alexander A. Osmolovskiy, Valeriana G. Kreyer

**Affiliations:** Faculty of Biology, Lomonosov Moscow State University, Moscow 119991, Russia

**Keywords:** *Aspergillus ochraceus* proteinase, protein C activators, factor X activators, fibrinolysis, fibrinogenolysis

## Abstract

The effect of *A. ochraceus* proteinase on the proteins of the human hemostasis system, fibrin, fibrinogen, plasminogen, protein C, and factor X, was studied. These proteins are key targets for proteolytic enzymes in therapy and diagnosis of thromboembolic complications. It was shown that *A. ochraceus* proteinase efficiently cleaves fibrin and fibrinogen, but does not act precisely, since it cuts all three subunits of these proteins. The proteinase did not have an activating effect on the plasminogen, a precursor of plasminogen and plasmin. The proteinase of *A. ochraceus* was shown to be the first fungal proteinase with proven activating activity towards the human hemostasis system factors protein C and factor X. For protein C activation, *A. ochraceus* proteinase requires Ca^2+^ ions. The enzyme was found to be sensitive to thrombin inhibitors, but not to plasmin inhibitors. A proteolytic action profile of the scope of this proteinase as a proteinase with activating protein C, factor X, and plasmin-like activity was proposed.

## 1. Introduction

The human hemostasis system is a complex set of reactions driven by a large number of proteins including proteolytic enzymes, either activating each other as a result of limited proteolysis or cleaving certain bonds, leading to proteolysis of target proteins. Such reactions can act as coagulants, anticoagulants, or fibrinolytics, altogether making up the corresponding parts of the hemostasis system. Regulation of the hemostasis system is carried out by the proteases themselves; the participants in the reactions and some of their inhibitors maintain a balance between blood coagulation and fibrinolysis. An imbalance of the system leads to the risk of thromboembolic complications and local bleeding [1,2]. Therefore, it is necessary to diagnose the activity of the components of the hemostasis system and, if necessary, subsequently correct it. Many tools intended for determining the activity of in vitro components of the hemostasis system and treating existing complications are proteolytic enzymes obtained both in recombinant form (human hemostasis proteases) and from other living organisms as exogenous proteins [3,4,5].

Many microscopic fungi—representatives of the genus *Aspergillus*—can produce proteolytic enzymes that are active against the proteins of the hemostasis system [6,7]. Their action can lead to both their proteolysis (hydrolysis) and activation through the reaction of limited proteolysis of proenzymes. *Aspergillus* proteases are known to exhibit a fibrinolytic effect, as well as to activate protein C, factor X, plasminogen, and prekallikrein. Their physicochemical properties are rather similar; however, the differences in substrate specificity enable considering them as promising agents for diagnostic medicine as exogenous activators of blood plasma proenzymes, as well as to expand the search for potential strains—producers of such enzymes [8,9].

*Aspergillus ochraceus* protease is a non-glycosylated protein with a molecular weight of about 35 kDa and pI 6.2, which has activating activity of protein C and X factor and also exhibits a plasmin-like effect. Its application to determine the content of the corresponding proenzymes of the hemostasis system was shown to be possible in *in vitro* tests [10]. Similar proteases included in commonly used diagnostic kits are obtained from the venom of the South American copperhead *Agkistrodon contortrix contortrix* (protein C activator) and the venom of Russell’s viper *Daboia russelli* (factor X activator). Unfortunately, due to the complex processing and post-translational modifications, these proteases are very costly to be cloned, so their native sources are still used. Therefore, the development of the *Aspergillus ochraceus* protease seems to be extremely important. This protease belongs to serine proteases with an observed optimal activity under a pH of 8.0–9.0 and 37 °C [10].

Of interest is the study of the action of *Aspergillus ochraceus* protease on target proteins of the hemostasis system.

The work aimed to determine the ability of *A. ochraceus* proteinase to hydrolyze and activate some blood plasma proteins, fibrin, fibrinogen, protein C, and factor X, and study some properties of the enzyme as a potential hemostatically active agent.

## 2. Results and Discussion

### 2.1. The Activity of A. ochraceus Proteinase against Proteins of the Hemostasis System and Its Proteolytic Action Profile of the Scope

A preparation of extracellular proteins was obtained from the culture liquid of the micromycete *A. ochraceus* by ammonium sulfate precipitation, followed by dialysis and freeze-drying. Consequently, fractions with pI 6.0–6.2 containing homogeneous proteinase were obtained using preparative isoelectric focusing of the preparation, as was shown earlier [10]. The study of the target proteolytic activity of the isolated proteinase with specific chromogenic peptide substrates of proteins of the hemostasis system and some plasma proteins made it possible to characterize it as an enzyme with an activating effect on protein C and factor X capable of proteolysis of both globular (casein) and fibrillar proteins (fibrinogen and fibrin).

The data obtained are presented in Table 1. As follows from the data given in the table, *A. ochraceus* proteinase cleaves chromogenic peptide substrates of activated protein C (pGlu-Pro-Arg-pNA), factor X (Z-D-Arg-Gly-Arg-pNA), and tissue plasminogen activator (H-D-Ile-Pro-Arg-pNA) in coupled reactions with human blood plasma and plasmin substrate H-D-Leu-Lys-pNA in direct reaction. Interestingly, the proteinase did not hydrolyze the tissue plasminogen activator substrate H-D-Ile-Pro-Arg-pNA directly. With regard to this fact, it was necessary to study the ability of proteinase to act directly on fibrin and fibrinogen. Determination of the corresponding types of activity showed that the proteinase hydrolyzes fibrinogen almost two times more actively than fibrin and cannot exert an activating effect on the plasmin precursor, plasminogen. In addition, it is obvious that proteinase actively hydrolyzes casein. The data obtained confirm the properties of the *A. ochraceus* proteinase [6] previously proposed and determine the proteolytic action profile of the scope. The proteolytic action profile of the scope (PAPS) should be understood as the minimum set of key substrates that determine the use of the protease in a particular area in relation to which it exhibits both its activator (due to limited proteolysis reactions) and hydrolytic activity. In the case of *A. ochraceus* proteinase, promising in the field of biomedicine, the PAPS can be defined as a proteinase with activating protein C, factor X, and plasmin-like activity. For the proteinase of another *Aspergillus*, *A. terreus*, the PAPS of the scope can be designated as a proteinase with activating prekallikrein and plasmin-like activity [9], and for *A. flavus*, it can be designated as a proteinase with activating plasminogen and plasmin-like activity [11].

### 2.2. Cleavage of Fibrin and Fibrinogen by A. ochraceus Proteinase

Plasmin-like activity includes hydrolyzing both fibrin and its precursor, fibrinogen. Thus, it was suggested to study this activity in detail by electrophoretic analysis of these substrates treated with *A. ochraceus* proteinase.

**Figure 1:**
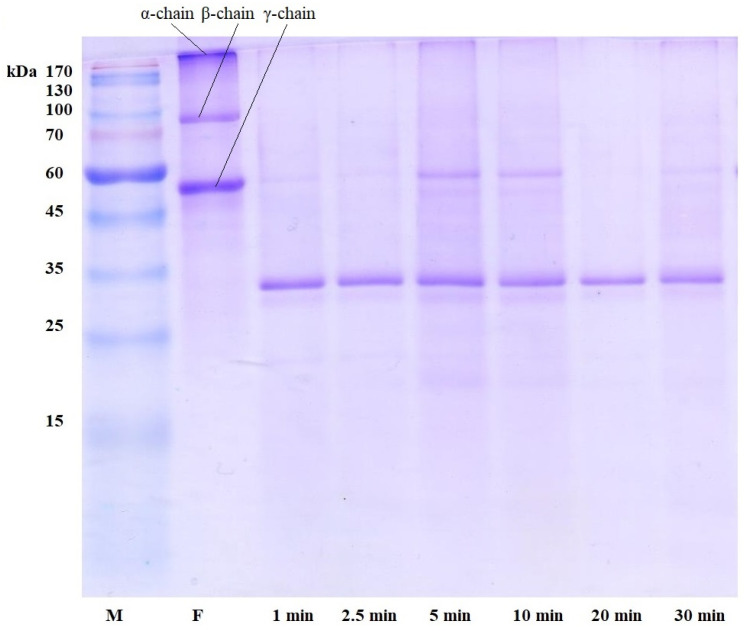
Proteolysis of fibrin by *A. ochraceus* proteinase. F, fibrin; M, molecular weight markers.

The fibrinolytic activity of *A. ochraceus* proteinase is expressed in the rapid destruction of α- and β-chains of fibrin for some time up to 1 min of incubation (Figure 1). The γ-chain, with a molecular weight of about 50 kDa, was not completely hydrolyzed; its protein fragment, which is small in concentration, is visible on the electrophoresis track after 30 min of incubation. Judging by the protein fragments from 15 to 45 kDa visible on the electrophoregram, proteolysis of fibrin by the *A. ochraceus* enzyme proceeds not to amino acids but to polypeptides of various lengths.

**Figure 2:**
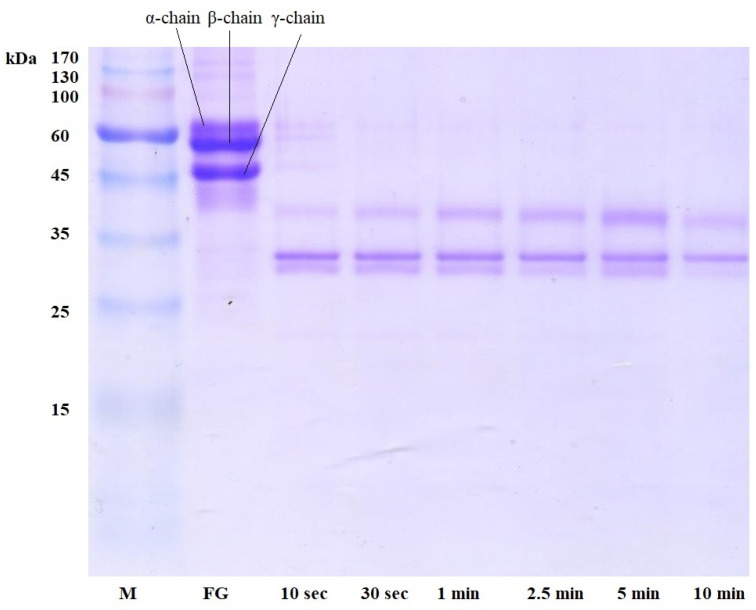
Proteolysis of fibrinogen by *A. ochraceus* proteinase. FG, fibrinogen; M, molecular weight markers.

The electrophoretic analysis of fibrinogen treated with *A. ochraceus* enzyme (Figure 2) was similar to the results of fibrinolysis: α-, β-, and γ-chains were completely hydrolyzed during the first 30 s of the reaction.

Similar, but not identical, results were observed during proteolysis of fibrin and fibrinogen by other extracellular *Aspergillus* proteinases—*A. alliaceus* 7dN1 and *A. terreus* 2—which have α-fibrinogenase, moderate β-fibrinogenase, and some γ-fibrinogenase activity [12].

The obtained results indicate a high affinity of *A. ochraceus* proteinase for fibrin and fibrinogen and a high degree of their hydrolysis, which can make this enzyme a promising blood-thinning and plasma-thinning agent in modern medical practice, especially in comparison with other fungal proteolytic enzymes. For example, it was shown that *A. alliaceus* 7dN1 proteinase hydrolyzed the α-chain of fibrin and fibrinogen completely, and the γ-chain partially hydrolyzed during fibrinolysis after the first 5 s; the degradation products of the γ-chain were completely hydrolyzed within 1 h [12]. Proteinases of some basidiomycetes can completely lyse fibrinogen and fibrin in up to 480 min [13].

### 2.3. Activation of Protein C and Factor X by A. ochraceus Proteinase

Of particular interest in the case of the properties and PAPS of *A. ochraceus* proteinase is its ability to activate the protein C and factor X of blood plasma. To confirm that an *A. ochraceus’* proteinase can activate protein C and human factor X, the test enzyme was incubated with these proteins.

**Figure 3:**
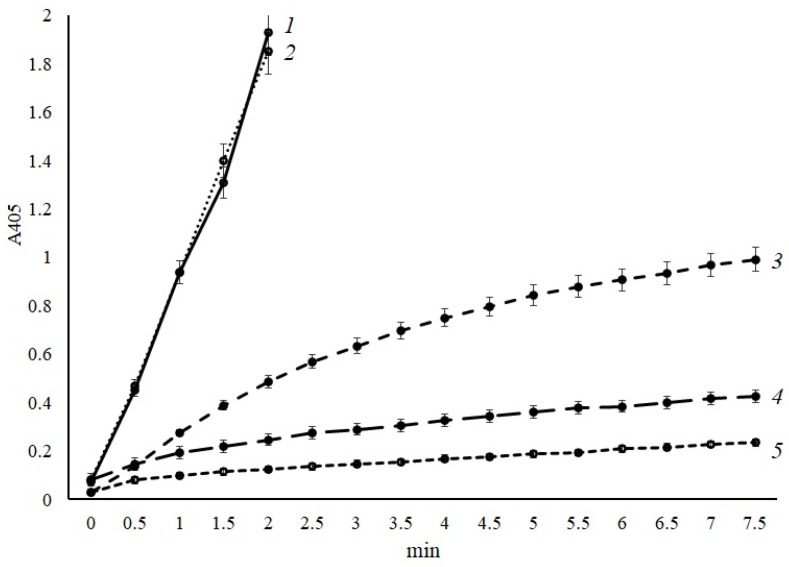
Effect of calcium ions on the activation of protein C by *A. ochraceus* proteinase: 1—5 × 10^−3^ M; 2—1 × 10^−2^ M; 3—2.5 × 10^−3^ M; 4—1 × 10^−3^ M; and 5—without the addition of calcium ions. Each point represents the mean of 3 measurements and the whiskers length is limited by the standard deviation. There were no outliers obtained in the experiment.

Determination of activity after preincubation of proteinase with protein C showed that it can indeed activate human protein C, but the development of the reaction proceeds very slowly over time (Figure 3). So, after 5 min of incubation with the substrate of activated protein C—pGlu-Pro-Arg-pNA—the specific activity was 9.8 U_pNA_/mg of protein × 10^−3^. Based on the obtained reaction kinetics, it was suggested that *A. ochraceus* proteinase needs a cofactor to accelerate the activation of protein C. Ca^2+^ ions can serve as such cofactors because their participation in the regulation of blood coagulation activity is well known. It is also known that different proteases, for their stability and activity, require Ca^2+^ ions [14].

So, the effect of calcium ions on protein C activation by the isolated enzyme was studied by adding a solution of calcium acetate of various concentrations to the reaction mixture. As can be seen from Figure 3, at a final concentration of Ca^2+^ ions of 1 × 10^−3^ M, there is a slight increase in the rate of activation of protein C by the *A. ochraceus* enzyme.

Increasing the concentration of calcium acetate by two times (up to 2.5 × 10^−3^ M) led to an increase in the rate of protein C activation, and the specific activity of the enzyme under these conditions was 73.4 U_pNA_/mg protein × 10^−3^, which is 7.5 times higher than the activation of protein C without Ca^2+^ ions (Figure 3).

At a concentration of calcium acetate in the incubation mixture of 5 × 10^−3^ M, the specific activity of the enzyme increased 25 times and amounted to 245.8 U_pNA_/mg protein × 10^−3^. Similar results were obtained at a concentration of calcium ions of 1 × 10^−2^ M.

Thus, it was confirmed that *A. ochraceus* proteinases are indeed capable of activating human protein C. Using this reaction as an example, limited proteolysis of blood plasma proteins by the proteolytic enzymes of this micromycete was established. The activation of protein C increases significantly in the presence of Ca^2+^ ions, which can serve as a cofactor for this reaction.

**Figure 4:**
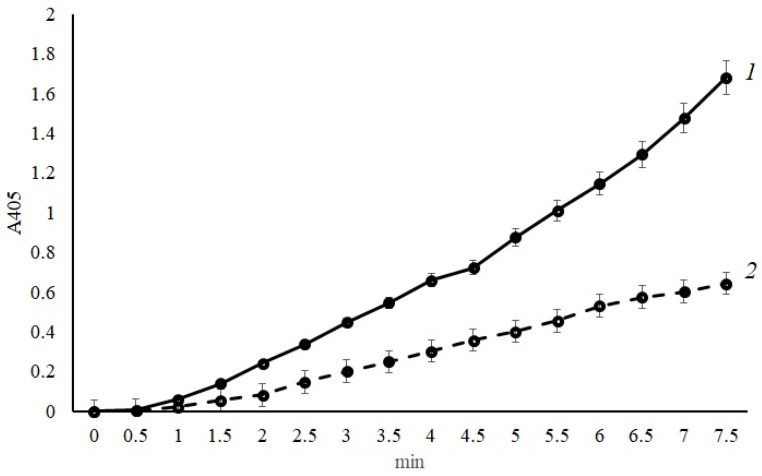
Factor X activation by *A. ochraceus* proteinase: 1—without calcium ions; 2—with the addition of calcium ions (1 × 10^−2^ M). Each point represents the mean of 3 measurements and the whiskers length is limited by the standard deviation. There were no outliers obtained in the experiment.

The study of human factor X activation by *A. ochraceus* proteinase was carried out both with Ca^2+^ ions and without (the concentration of 1 × 10^−2^ M was used immediately). The reaction’s kinetics were determined with the substrate of activated factor X—Z-D-Arg-Gly-Arg-pNA. The obtained data are shown in Figure 4. As illustrated, *A. ochraceus* proteinase is also capable of factor X activation, however, without calcium ions in the incubation mixture (4.6 U_pNA_/mg protein × 10^−3^). The addition of calcium ions not only had no stimulating effect on the development of the activation reaction but also reduced its rate by 2.5 times (1.8 U_pNA_/mg protein × 10^−3^).

### 2.4. Kinetic Studies of Plasmin-like Activity of Proteinase A. ochraceus

**Figure 5:**
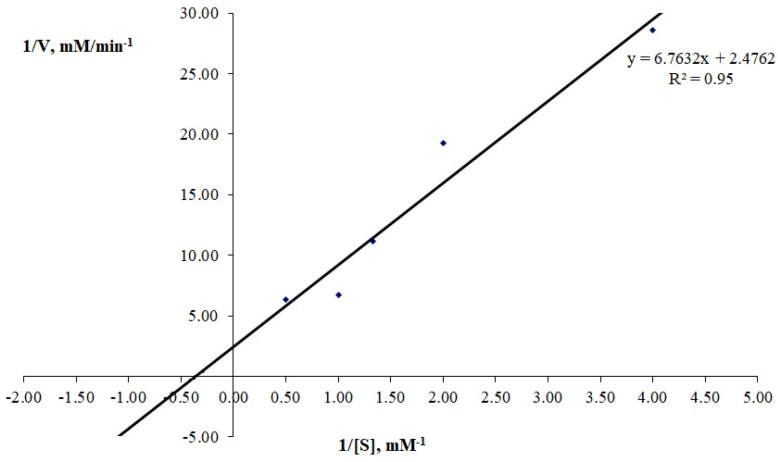
Lineweaver–Burke coordinates for determination of kinetic parameters of *A. ochraceus* proteinase. Each point represents the mean of 3 measurements.

The kinetic parameters K_m_ and V_max_ of the enzyme were determined with the chromogenic plasmin substrate H-D-Leu-Lys-pNA and corresponded to 3.48 mM and 4.17 mM/min, respectively (Figure 5). For comparison, it can be noted that they differed from the similar parameters of this enzyme determined previously with the chromogenic thrombin substrate Tos-Gly-Pro-Arg-pNA, which were 2.34 mM and 1.66 mM/min, respectively [10].

### 2.5. Hemostatic Inhibitors Effect

**Figure 6:**
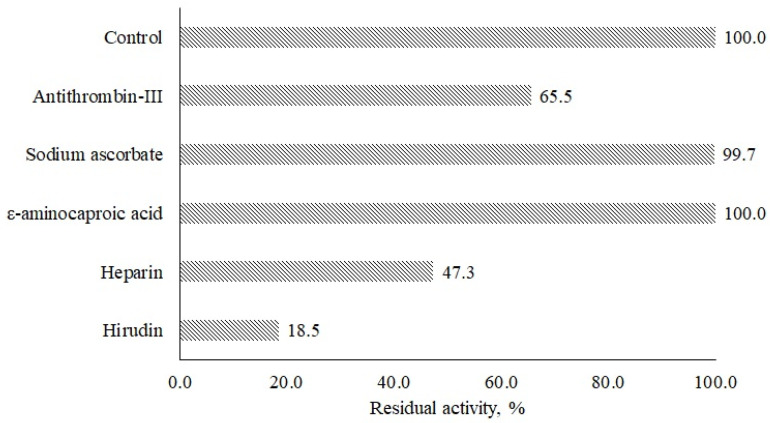
Effect of thrombin and plasmin inhibitors on the activity of *A. ochraceus* proteinase.

In the last step, the effect of natural inhibitors of hemostatic factors on *A. ochraceus* protease activity (Figure 6) was analyzed. It was found that inhibitors of thrombin like antithrombin III, heparin, and hirudin decreased the proteolytic activity of the enzyme by 34.5, 52.7, and 81.5%, respectively. ε-aminocaproic acid and sodium ascorbate as inhibitors of plasmin did not affect the proteolytic activity under experimental conditions. Similar results were obtained when the properties of *A. terreus* proteinase were studied. 

## 3. Materials and Methods

### 3.1. A. ochraceus Proteinase Preparation

In this research, we used the extracellular proteinase of the micromycete *Aspergillus ochraceus* from the collection of the Department of Microbiology, M.V. Lomonosov Moscow State University, obtained as described earlier [5]. The strain was cultivated under submerged conditions in orbital shaker ES-20/60 (“Biosan”, Rīga, Latvia) at 200 rpm and 28 °C in two consecutive stages, with growth on seed (composition in %: wort—6.7; glucose—1.0; and peptone—0.1, pH 5.5–6.0) and fermentation media (composition in %: glucose—3.5; fish flour hydrolysate—1.0; NaCl—0.2; starch—0.125; peptone—0.1; KH_2_PO_4_—0.05; and MgSO_4_—0.05, pH 5.5–6.0). The inoculum obtained via spore flushing with the seed medium was then introduced into the seeding medium and cultured for 2 days. Part of the biomass was then transferred to the fermentation medium and cultured for another 2 days. The cultivation was carried out in 750 mL shake flasks containing 100 mL of culture medium [9]. The preparation of extracellular proteinases of the micromycete was obtained by ammonium sulfate precipitation (608 g (NH_4_)_2_SO_4_ per 1 L) of the culture liquid filtrate. Precipitated proteins were collected after centrifugation with cooling at 15,000× *g* for 20 min at 4 °C. The resulting precipitate was dissolved in 0.01 M Tris-HCl buffer (pH 8.2) and dialyzed in dialysis tubes against the same buffer at 4 °C for 18 h. The dialyzed preparation was frozen with liquid nitrogen and lyophilized. Fractionation of the obtained preparations (25 mg/mL) was carried out using preparative isoelectric focusing according to the Vesterberg method in a pH gradient of ampholines 2.5–10.0 and a sucrose density gradient of 0–40% in a 110 mL column (LKB, Luleå, Sweden) at a voltage of 800 V for 36 h [6]. 

### 3.2. Proteolytic Assays

For the study of proteolysis of native substrates, 1% (*w*/*v*) suspensions of Hammerstein’s casein (“Sigma-Aldrich”, Saint Louis, MO, USA), bovine fibrinogen (“Sigma-Aldrich”, USA), and bovine fibrin (“Sigma-Aldrich”, USA) were used. The activities of *A. ochraceus* proteinase were determined by Anson–Hagihara’s modified method [15]. For the reaction, 200 μL of the sample and 400 μL of suspension of the corresponding protein substrate (in 0.1 M Tris-HCl buffer (pH 8.2)) were mixed. After that, the mixtures were incubated for 10–30 min at 37 °C with permanent shaking (600 rpm). The reactions were stopped by adding 600 μL of 10% trichloroacetic acid. Then, samples were centrifugated (12,400× *g*, 10 min), and A275 was measured in supernatant. The activity was expressed in μmoles of tyrosine formed in 1 min in 1 mL of culture liquid (U_Tyr_).

Fibrinolytic and plasminogen activating activities were determined using the fibrin plates method according to Astrup–Mullertz–Lassen [16]. To prepare a fibrin plate, 9 mL of bovine fibrinogen solution (0.76%) and 0.2 mL of thrombin (0.4%) were mixed in a Petri dish. To dissolve the proteins, a mixture of physiological saline and 0.05 M Tris-HCl buffer (pH 8.2) was used in a ratio of 9:1. Petri dishes with the fibrin plates to determine fibrinolytic activity were heated in a drying chamber oven at 86 °C for 30 min to inactivate the plasminogen impurity. Dishes with fibrin plates for determination of activator activity to plasminogen were not warmed up. A total of 30 μL of the sample was added to the surface of the fibrin plates, after which the dishes were put in a thermostat for 3 h at 37 °C. The activity was expressed in arbitrary units (a.u.) per 1 mL of the sample. One arbitrary unit of activity was taken as the amount of enzyme causing the zone of fibrin lysis equal to 10 mm^2^.

Plasmin-like and t-PA-like (tissue plasminogen activator) activities were measured with chromogenic peptide substrates (“Chromogenix”, Milan, Italy) H-D-Leu-Lys-pNA and H-D-Ile-Pro-Arg-pNA, correspondingly. For the reaction, 50 µL 0.05 M Tris-HCl buffer, pH 8.2, and 100 μL of a 0.05% (0.05 M Tris-HCl buffer, pH 8.2) solution of appropriate chromogenic peptide substrate were added to 200 µL of the enzyme solution (0.2 mg/mL), and incubated for 5 min at 37 °C. The reaction was stopped with 200 μL of 50% acetic acid [4].

For the determination of protein C-activating and factor X-activating activities, chromogenic peptide substrates (pGlu-Pro-Arg-pNA and Z-D-Arg-Gly-Arg-pNA, correspondingly) were used (“Chromogenix”, Italy). For reactions, 50 µL of a human plasma sample (diluted 2 times in 0.05 M Tris-HCl buffer, pH 8.2) was added to 200 µL of the enzyme solution (0.2 mg/mL) and preincubated for 5 min at 37 °C. After that, a solution of the corresponding substrate was added to 100 μL of a 0.05% buffer (0.05 M Tris-HCl buffer, pH 8.2), and the reaction was continued under the same conditions for 5 min. The reaction also was stopped with 200 μL of 50% acetic acid [9]. The absorbance of emitted pNA under 405 nm was measured.

The unit of activity (U_pNA_) was the amount of µmol of p-nitroaniline cleaved from the chromogenic substrate in 1 min.

### 3.3. Protein C and Factor X Activation by the Protease of A. ochraceus 

The ability to activate protein C by *A. ochraceus* proteinase was determined using pure human protein C (“Sigma-Aldrich”, USA). A total of 50 µL of a solution of pure human protein C (4 mg/mL) in 0.05 M Tris-HCl buffer, pH 8.2, was taken into the reaction. The reaction was carried out with a proteinase solution (0.2 mg/mL). Preincubation with protein C was carried out with 100 µL of the proteinase of *A. ochraceus* and 100 µL of 0.05 M Tris-HCl buffer, pH 8.2. The buffer either contained no calcium acetate or contained it to adjust final concentrations of calcium ions in the incubation mixture to 1 × 10^−3^ M, 2.5 × 10^−3^ M, 5 × 10^−3^ M, and 1 × 10^−2^ M. Optical density at 405 nm of emitted pNA was determined spectrophotometrically.

To determine the ability to activate factor X by *A. ochraceus* proteinase, the human factor X (4 mg/mL, “Sigma-Aldrich”, USA) was used. The reaction was carried out in the same conditions as for the protein C activation. The activity was measured with chromogenic peptide substrate Z-D-Arg-Gly-Arg-pNA with the presence or absence of calcium acetate (1 × 10^−2^ M).

### 3.4. Protein Content Determination

Protein content was determined by the Bradford protein assay [17]. Specifically, 950 μL of the Coomassie Brilliant Blue G-250 reagent was added to 50 μL of the sample, and A595 was recorded. The protein concentration was calculated by performing a Bradford calibration curve.

### 3.5. Electrophoretic Studies of Fibrinogen and Fibrin Cleavage

Fibrin and fibrinogen were cleaved by proteinase and subsequently analyzed by SDS-PAGE [18]. For the reaction, 50 µL of a 0.1% solution of fibrin or fibrinogen (“Sigma-Aldrich”, USA), prepared in 0.1 M Tris-HCl buffer, pH 8.2, was added to 25 µL of a sample containing proteinase and incubated a certain time (in the range from 10 s to 60 min) at 37 °C with constant stirring (600 rpm). The reaction was stopped with 25 µL of sample buffer prepared in 0.00625 M Tris-HCl buffer, pH 6.8, containing the following (%): SDS—2.0; sucrose—10.0; mercaptoethanol—5.0; and bromophenol blue—0.001. After stopping the reaction, the samples were kept at a temperature of 100 °C for 3 min and applied to the gel for electrophoresis. Electrophoresis was carried out in PAAG under denaturing conditions according to the Laemmli method at a current strength of 20 mA [18].

### 3.6. Kinetic Studies

Kinetic parameters such as maximum reaction rate (V_max_) and Michaelis constant (K_m_) were determined for plasmin-like activity with the chromogenic peptide substrate H-D-Leu-Lys-pNA as described above. The substrate was used at concentrations of 0.25, 0.5, 0.75, 1.0, and 2.0 mg/mL.

The reaction kinetics were recorded automatically using a spectrophotometer under 405 nm absorbance reading. The dependence of the reaction rate on the concentration of substrates was plotted in the Lineweaver–Burke and Eady–Hofstee coordinates.

### 3.7. Effect of Plasma Proteins Inhibitors on Protease of A. ochraceus Activity

The effect of natural inhibitors of hemostasis system proteins on *A. ochraceus* proteinase (0.25 mg/mL) was studied. Hirudin (1.5 mg/mL, “Sigma-Aldrich”, USA) and antithrombin III (1.5 mg/mL, “Renam”, Moscow, Russia) were used as thrombin inhibitors because they slow down or completely stop the activation of thrombin by coagulation factors V, VIII, and XIII: heparin (5.3 mg/mL, “Renam”, Russia) as an inhibitor of thrombin and coagulation factors IXa, Xa, XIa, and XIIa; ε-aminocaproic acid (1.3 mg/mL, “Roth”, Nuremberg, Germany) as an inhibitor of plasmin and tissue plasminogen activator; and sodium ascorbate (1.75 mg/mL, “Roth”, Germany) as an inhibitor of blood coagulation.

The residual activity was determined with the chromogenic peptide substrate H-D-Leu-Lys-pNA, as mentioned above, after 1.5 h of preincubation of the enzyme and inhibitor at room temperature and was expressed as a percentage of control one (reaction without inhibitor). 

All reactions were carried out in a TS-100 thermoshaker (“BioSan”, Latvia). The optical density of the solutions was measured on an Eppendorf kinetics spectrometer (“Eppendorf”, Hamburg, Germany).

The experiments were carried out in three replicates, with the error not exceeding 5–7%. The data were statistically processed using MS Excel 2019 and Statistica 7.0. The Mann–Whitney U test was used to compare the data; differences were considered statistically significant at *p* < 0.05.

## 4. Conclusions

Thus, the effect of *A. ochraceus* proteinase on the proteins of the human hemostasis system, fibrin, fibrinogen, plasminogen, protein C, and factor X, was studied. These proteins are key targets for the action of proteolytic enzymes in the therapy and diagnosis of thromboembolic complications. It was shown that *A. ochraceus* proteinase actively hydrolyzes fibrin and fibrinogen, but does not show a target action, since it hydrolyzes all three subunits of these proteins indiscriminately. Proteinase did not have an activating effect on the plasminogen, a precursor of plasmin. The proteinase of *A. ochraceus* was shown to be the first fungal proteinase with proven activating activity towards the human hemostasis system factors protein C and factor X. For protein C activation by proteinase of *A. ochraceus,* Ca^2+^ ions are required. The proteinase was found to be sensitive to thrombin inhibitors, but not to plasmin inhibitors. A proteolytic action profile of the scope of this proteinase as a proteinase with activating protein C, factor X, and plasmin-like activity was proposed. This study of a bi-functional proteinase - with activator and fibrino(geno)lytic activity - contributes to the biotechnological potential of the *Aspergillus ochraceus* species, confirming its significance [19].

## Figures and Tables

**Table 1:** Proteolytic activity of *Aspergillus ochraceus* protease.

Activity	Units of Activity	Value
Protein C-activating	U_pNA_/mg	165.3
Factor X-activating	U_pNA_/mg	88.9
Plasmin-like	U_pNA_/mg	134.8
t-PA-activating	U_pNA_/mg	87.2
t-PA-like	U_pNA_/mg	0.0
Caseinolytic	U_Tyr_/mg	701.6
Fibrinolytic	U_Tyr_/mg	218.0
Fibrinogenolytic	U_Tyr_/mg	514.5
Fibrinolytic	a.u./mg	2405.0
Plasminogen-activating	a.u./mg	0.0

## Data Availability

Not applicable.
